# Physical Literacy as a Pedagogical Model in Physical Education

**DOI:** 10.3390/children12081008

**Published:** 2025-07-31

**Authors:** Víctor Manuel Valle-Muñoz, María Mendoza-Muñoz, Emilio Villa-González

**Affiliations:** 1 Department of Physical Education and Sports, Faculty of Sport Sciences, Sport and Health University Research Institute (iMUDS), University of Granada, 18071 Granada, Spain; evilla@ugr.es; 2 Research Group on Physical and Health Literacy and Health-Related Quality of Life (PHYQOL), Faculty of Sport Sciences, University of Extremadura, 10003 Caceres, Spain; mamendozam@unex.es

**Keywords:** physical literacy, physical education, pedagogical model and physical activity

## Abstract

**Background/Objectives**: Legislative changes in educational systems have influenced how student learning is understood and promoted. In physical education (PE), there has been a shift from behaviorist models to more holistic approaches. In this context, physical literacy (PL) is presented as an emerging pedagogical model in school PE, aimed at fostering students’ motor competence in a safe, efficient, and meaningful way. The aim of this study is to analyze the origins, foundations, methodological elements, and educational value of PL, highlighting its potential to promote holistic and inclusive learning as the basis for an emerging PL model. **Methods**: A narrative review was conducted through a literature search in the Web of Science, PubMed, Scopus, and SportDiscus databases up to June 2025, focusing on scientific literature related to PL and PE. The analysis included its historical background, philosophical and theoretical foundations, and the key methodological elements and interventions that support its use as a pedagogical model. **Results/Discussion**: The findings indicate that the PL model can be grounded in key principles, such as student autonomy, teacher training, connection with the environment, inclusion, and collaboration. Additionally, motivation, enjoyment, creativity, and continuous assessment are identified as essential components for effective implementation. Moreover, this model not only guides and supports teachers in the field of PL but also promotes comprehensive benefits for students at the physical, cognitive, affective, and social levels, while encouraging increased levels of physical activity (PA). **Conclusions**: PL is understood as a dynamic and lifelong process that should be cultivated from early childhood to encourage sustained and active participation in PA. As a pedagogical model, PL represents an effective tool to enhance student learning and well-being in PE classes.

## 1. Introduction

The educational reform in Spain during the 1990s marked a shift toward a holistic view of student development, emphasizing cognitive, affective, relational, and social dimensions [1,2]. This transition from a behaviorist to a constructivist model redefined ability as the capacity to engage with diverse contexts, forming the basis for developing competencies [3,4]. Physical education (PE) was part of this change, introducing new methodologies and responding to declining motivation for physical activity (PA) with age [5,6,7]. Currently, over 80% of adolescents aged 11–17 do not meet global PA recommendations [8], with similar data reported by the Global Matrix 4.0, where only 27–33% meet the suggested daily levels [9]. In Spain, 76.6% of youth are inactive, especially girls [10].

Therefore, methodological approaches in PE developed alongside Mosston’s teaching styles from the 1960s [11], which remain influential today. In Spain, Delgado [12] adapted Mosston’s framework, now widely used in universities and PE classes. Over time, various pedagogical models also emerged in Spain [13], leading to evolving terminology, such as Teaching Models [14], Curriculum Models [15,16], Instructional Models [17], and ultimately Pedagogical Models [18]. These emphasize the interplay of learning, teaching, content, and context [19], though their evolution has generated educational debates [20,21,22]. Flórez [23] views them as interconnected teaching–learning parameters, requiring a solid theoretical grasp for effective application [17]. Others note that models are dynamic, evolving with new knowledge [24], and often build upon earlier principles [25]. Since the 1970s, multiple pedagogical models have emerged both in the field of sport and in PE more broadly, such as Cooperative Learning [26,27], Sport Education [28,29], Teaching Game for Understanding [30,31], or the Personal and Social Responsibility Model [32,33]. However, in the effort to develop new pedagogical approaches, the so-called emerging models have appeared [13], such as the Ludotechnical model [34,35], Self-constructed Equipment [36], Health Education [37], the Attitudinal Style [38], or Physical Literacy (PL) [39], which is addressed in the development of this article.

PL is addressed in the development of this article as one of the most prominent emerging models, recognized for its potential to contribute meaningfully to the holistic development of students through PE [39]. Defined as the integration of physical competence, motivation, confidence, and understanding to engage in lifelong PA [40], PL has gained international attention and is now a cornerstone in major frameworks, such as the WHO Global Action Plan on PA 2018–2030, UNESCO’s Guidelines on Quality PE, and Spain’s latest education reform (LOMLOE) [41,42,43,44]. Whitehead [39,45] argues that PL should not be viewed as a rival to PE but as its ultimate educational aim, offering a broader and more inclusive vision of human movement. PL encompasses a holistic and lifelong process that integrates physical, cognitive, affective, and social domains, empowering individuals to engage in meaningful and contextually relevant PA [46]. Consequently, institutions, such as SHAPE America and UNESCO, define quality PE as that which nurtures physically literate individuals, capable of sustaining active lifestyles across the lifespan [47,48].

Various national education systems have embraced this vision. Countries, like Canada and Australia, have led in integrating PL into curricular policies, the latter through its comprehensive Australian Physical Literacy Framework (APLF), which articulates 30 interconnected elements across multiple domains [49,50,51,52]. Similarly, several UK regions and the Spanish LOMLOE have aligned with this paradigm, reinforcing inclusive and integrative PE [43,48,53,54,55]. Despite its increasing adoption, PL remains conceptually and philosophically contested. Over the past two decades, scholars have debated its definitional clarity, theoretical foundations, and practical implementation [56,57,58,59]. Some argue that PL risks becoming more of a persuasive slogan than a rigorously defined construct, due to its inconsistent application across research, policy, and practice [60,61]. The coexistence of overlapping and sometimes contradictory definitions—from metaphorical interpretations to reductions based on technical skills or digital platforms [62,63]—adds to this ambiguity. The proliferation of related constructs (e.g., health literacy, movement literacy, games literacy) also dilutes the term’s distinctiveness [64]. Two dominant paradigms have shaped PL’s evolution: the Whiteheadian model, grounded in phenomenology, monism, and existentialism [59], and the Long-Term Athlete Development (LTAD) model, originally focused on physical capacities for sport performance [64]. Although recent adaptations of LTAD attempt to expand its educational scope [65], it remains centered on physical outcomes, which some argue can be incorporated into the broader Whiteheadian framework—but not vice versa [44].

This divergence highlights the need for clearer epistemological grounding and theoretical articulation. Without transparent assumptions or testable mechanisms, PL risks losing academic credibility [58]. Additionally, scholars warn against framing PL as a universal educational ideal, noting that such narratives may marginalize culturally diverse pedagogies and reproduce dominant norms [66]. A more context-sensitive, pluralistic perspective—one that adapts PL to the institutional, cultural, and social realities of each educational context—could offer a more productive path forward. Within this complex landscape, educational researchers advocate for refining the concept of PL in PE to support not only the acquisition of motor skills but also the development of body language as a communicative tool in social and cultural contexts [67,68]. A recent systematic review by Pereira and Catalán [69] reinforces this by proposing a framework to guide quality innovation in PE, calling for an ecological approach that integrates institutional leadership with classroom practice. For PL to fulfill its promise, it must be underpinned by rigorous theoretical foundations, remain open to critical scrutiny, and address the multifaceted realities of contemporary PE.

In this context, the present study is justified by three key factors: (1) the growing international interest in PL as a conceptual framework to rethink PE, contrasted with the current lack of concrete pedagogical models for its implementation [70,71,72]; (2) the epistemological and curricular challenges teachers face when attempting to translate PL’s philosophical underpinnings into practice—especially in culturally diverse educational contexts; and (3) the pressing need for professional development programs that enable educators to understand, apply, and adapt PL principles effectively and meaningfully in their teaching [48,73]. Although PL is widely promoted as an inclusive and holistic educational goal, its successful integration into school settings depends largely on teachers’ capacity to incorporate its principles into everyday practice. Therefore, robust and well-structured teacher training becomes not only valuable but essential. In response to these challenges, and despite ongoing debates, there is increasing consensus that PE should offer rich, varied, and meaningful experiences that support the development of PL. This article, therefore, proposes a pedagogical model aimed at guiding its practical implementation in educational settings. The model seeks to equip educators with tools to foster physically literate students capable of engaging in lifelong PA.

Therefore, the article aims to explain and justify the foundations that support PL as an emerging pedagogical model in PE. Several scholars have called for a models-based approach to PE pedagogy [72] and for the development of a coherent framework grounded in PL [20,74,75]. Following the structural and methodological approaches of Pérez-Pueyo et al. [38] and Gleddie and Morgan [76], this study adopts a theoretical–conceptual perspective, grounded in critical reflection and narrative literature review rather than empirical methods. Based on the growing international recognition of PL and the pedagogical vacuum surrounding its implementation, this article examines the philosophical, theoretical, and pedagogical principles that underpin its integration into educational practice. The main objective of this work is to justify PL—supported by strong philosophical foundations and emerging empirical evidence—as a viable, inclusive, and coherent pedagogical model capable of promoting holistic and lifelong learning through quality PE.

## 2. Methods

To address the objective of this study, a narrative review was conducted using a systematized strategy that enabled rigorous exploration, analysis, and synthesis of the existing literature on PL as an emerging pedagogical model in PE. Although qualitative and reflective in nature, this methodology is based on structured search criteria that ensure the validity and relevance of the reviewed content. It follows the methodological guidelines proposed by Pérez-Pueyo et al. [38] and Gleddie and Morgan [76], who have developed theoretical PE models through critical and well-founded literature analysis. Additionally, the review process adheres to the PRISMA guidelines for transparent and comprehensive reporting of reviews [77].

### 2.1. Search Strategy

For study identification, the academic databases Web of Science, PubMed, Scopus, and SportDiscus were systematically consulted, selecting works that addressed PL from an educational or pedagogical perspective. The search terms used were “Physical Literacy” and “Physical Education,” which enabled the identification of conceptual, theoretical, and empirical studies analyzing the development, application, or philosophical foundations of PL in educational contexts. No temporal limit was applied in the initial search up to 3 June 2025, as it was essential to consider both foundational and recent contributions in order to justify the origins, philosophical principles, and evolving conceptual frameworks of PL. This broad timeframe allowed for a more complete and rigorous understanding of the term’s progression. In addition, gray literature—including policy documents, institutional reports, and academic books—was also consulted to reinforce the theoretical justification and practical applicability of PL across diverse educational and cultural settings.

### 2.2. Inclusion and Exclusion Criteria

The selection process followed PRISMA guidelines [77] and included peer-reviewed publications written in English or Spanish that explicitly addressed PL as a conceptual and pedagogical framework within the context of PE. Eligible studies focused on school-aged children and adolescents (5–17 years old) and encompassed various methodological designs, including systematic reviews, theoretical essays, and both quantitative and qualitative research. Studies were included if they: (i) presented a pedagogical, curricular, or conceptual analysis of PL aligned with recognized frameworks—such as the Australian APLF, the Physical Literacy in Practice (PLP) model, or the International Physical Literacy Association (IPLA) definition; (ii) explored the integration or interpretation of PL in PE contexts; and (iii) contributed to the theoretical or practical understanding of PL in educational settings. Excluded were publications that (i) focused on non-school contexts (e.g., clinical, extracurricular, or community settings), (ii) targeted populations outside the specified age range, or (iii) consisted solely of conference abstracts, posters, or unpublished theses.

### 2.3. Selection and Extraction

**Figure 1:**
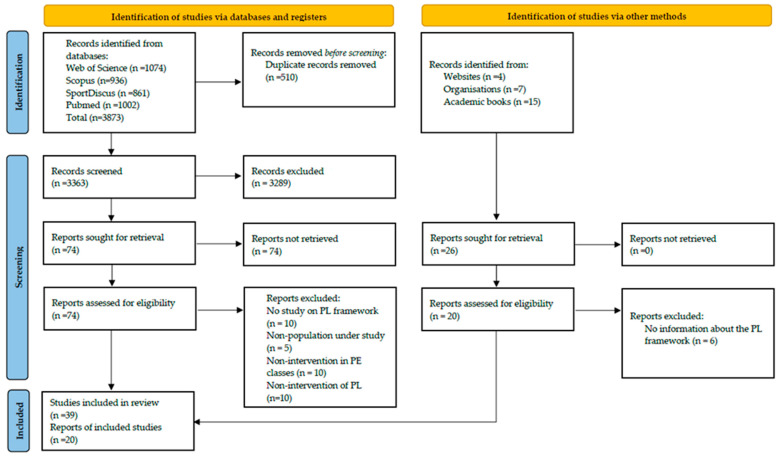
PRISMA flow diagram of literature search.

The selection process was conducted systematically following the PRISMA [77] flow diagram guidelines. In the identification phase, the first author (VM) screened all titles and abstracts to exclude studies that were clearly irrelevant based on the predefined inclusion criteria. To ensure greater consistency, 10% of the records were independently cross-checked by a second reviewer (MM). Studies identified as potentially eligible were then retrieved in full text and independently reviewed by both authors (VM and MM). During this phase, each article was carefully analyzed, and any disagreements regarding inclusion were resolved through discussion until consensus was reached. All references and records related to the selection process were managed using EndNote version 9.0 to minimize potential selection and reporting bias. Data extraction was carried out by one reviewer (VM) and independently verified by a second reviewer (MM) to ensure accuracy and consistency in the extracted information. When necessary, the authors of the original studies were contacted to obtain missing information. Extracted data included the philosophical origins of PL, related pedagogical interventions, and the key components that constitute its possible theoretical framework. During the identification phase, a total of 3873 studies were retrieved (Figure 1). After removing 510 duplicates, 3363 articles were screened by title and abstract in the selection phase. Of these, 3289 were excluded due to reasons, such as lack of thematic relevance, target populations outside the defined age range, absence of PL-based interventions, or failure to directly address the concept of PL. In the eligibility phase, 74 full-text articles were assessed, with 35 subsequently excluded based on predefined criteria. In the final inclusion phase, 39 articles met all inclusion criteria and were included in the final review. Additionally, 20 relevant sources from grey literature were included, identified through Google Scholar, encompassing institutional websites, organizational documents, and key books that contributed to the consolidation of the theoretical framework of the review.

In addition, it is important to highlight that various types of studies were included, among them theoretical articles, systematic reviews, and experimental studies that evaluated specific PL interventions in PE classes. These studies involved more than 1500 participants, including children and adolescents from diverse educational contexts. Regarding the theoretical frameworks adopted, three main approaches were integrated: the health framework, which conceives PL as a key factor in physical well-being and disease prevention; the “Physical Literacy Praxis” model by Gleddie and Morgan [76], which offers a pedagogical perspective centered on educational praxis; and, notably, the APLF, currently considered the most comprehensive and up-to-date model for supporting pedagogical proposals aligned with the principles of PL. Additionally, key academic books were included to deepen the understanding of the origins, philosophical development, and conceptual foundations of PL—elements essential to theoretically support our proposed pedagogical model.

## 3. Results

Based on the results obtained, the review was structured around five analytical axes considered essential to justify PL as an emerging pedagogical model. First, the origin and historical evolution of the concept of PL were examined in order to understand its philosophical roots and conceptual trajectory. Second, the philosophical foundations underpinning PL were explored, with a particular focus on the traditions of monism, existentialism, and phenomenology. These offer an ontological and epistemological framework that views the individual as an integrated and situated being, constantly interacting with the world. Third, contemporary conceptual frameworks linking PL to education and health were analyzed, emphasizing its connection to PA and health. This perspective supports the argument that PL is not only relevant within the classroom but also serves as a key driver for promoting active, sustainable, and healthy lifestyles. Fourth, pedagogical elements necessary for fostering the development of PL in PE classes were identified and discussed. Finally, the fifth axis focused on reviewing various PL-based interventions implemented in school PE settings, evaluating their approaches, methodologies, and outcomes. These five thematic blocks form the foundation of the present study and support the development of a pedagogical model grounded in PL—designed to guide educational interventions in PE through a holistic, inclusive, and transformative lens.

### 3.1. Origin of Physical Literacy

The concept of literacy has been present throughout history and has been understood as “being educated or cultured” [78], implying the ability to identify, understand, interpret, create, and communicate information in different contexts [47]. In education, this notion has evolved and been applied to various domains, such as digital, nutritional, numerical, or health literacy [79]. PL has been present in PE since the 1920s. Franklin Bobbitt, in his work Objectives of Physical Education, mentioned the importance of knowledge about exercise skills, though without explicitly linking the term literacy to the physical domain [47]. Later, in 1930, James Edward Rogers, director of the National PE Service of the U.S. Association of Games and Recreation, stated that “public schools are responsible for physical and mental literacy” [80], though he did not precisely define the concept.

In 1942, Jesse Williams introduced the idea of the “new PE” in his book Principles of Physical Education, in which he advocated for the holistic development of the individual through PA [81]. His notion of “education through the physical” laid the foundation for the conceptualization of PL [82]. Later on, Charles McCloy was the first academic to specifically introduce the term physical or motor literacy in PE. In his research, he used both terms interchangeably, emphasizing that students could develop these skills through the mechanical improvement of their motor abilities in PE classes [83]. During the 1960s, Morrison expanded the perspective of PL by defining a physically literate individual as someone who can perform efficient, creative, and competent movements with enthusiasm [84]. Similar concepts emerged in the 1960s and 70s, such as “kinesthetic intelligence,” “intelligent action,” and “skilled action” [85]. Later, in 1991, the Sports Council of the United Kingdom introduced the term “movement literacy” in a brochure aimed at raising awareness about sports literacy as a concept parallel to traditional literacy [86]. The modern definition of PL was proposed by Margaret Whitehead in 1993, stating that a physically literate person should “move with poise, economy, and confidence in a wide range of physically challenging situations” and be able to interpret their environment, anticipating and responding appropriately to movement demands with intelligence and imagination [82]. A recent study identified nine definitions of PL across different time periods and international contexts, relating them to concepts, such as education, PA, health, competence, and curriculum [87]. From this analysis, 22 distinct definitions of PL have been identified, highlighting the seminal formulation by Whitehead [39], which describes PL as “the disposition to capitalize on our embodied human capability, in which the individual has the motivation, confidence, physical competence, knowledge, and understanding to value and take responsibility for engaging in purposeful physical pursuits throughout the life course.” This definition underscores a holistic approach that extends beyond mere physical skills to incorporate affective and cognitive dimensions. Consistent with this, in recent years, researchers, practitioners, and policymakers have increasingly debated the concept of PL [88], emphasizing its role in promoting lifelong physically active lifestyles by integrating multiple individual determinants of PA [87]. These determinants commonly encompass physical, affective, cognitive, and occasionally social or spiritual factors [87], depending on the specific definition adopted [58]. Among the most widely recognized conceptualizations, the International Physical Literacy Association (IPLA), building on Whitehead’s foundational ideas, defines PL similarly as “the motivation, confidence, physical competence, knowledge, and understanding to value and take responsibility for engaging in physical activities for life” [45]. Complementing this, the APLF articulates PL as “holistic lifelong learning acquired and applied in movement and PA contexts, reflecting ongoing changes integrating physical, psychological, social, and cognitive capacities” [52]. More recently, China’s definition emphasizes PL as “the integration of physical, perceptual, cognitive, psychological, and behavioral capacities aligned with the need for an active, healthy, and satisfying lifestyle, involving continuous positive interactions with the environment and embodied participation in lifelong physical activities” [89]. Sport England also contributes a unique interpretation, describing PL as the “individuals’ personal relationship with movement and PA throughout life” [90].

Although there are nuanced differences in the emphasis of particular domains—such as social elements in the Australian model or perceptual aspects in the Chinese framework—the original conceptualizations of PL universally rest upon established philosophical assumptions about human life. These include the inseparability of body and mind (monism/embodiment), connection with the environment (existentialism), and the authenticity of individual perceptions and life narratives (phenomenology) [67]. Such philosophical foundations challenge unilateral intervention approaches and the prioritization of single determinants (e.g., purely psychomotor aspects) [17]. Indeed, PL distinctly distances itself from external performance standards, instead favoring a person-centered perspective on PA [65]. It is also important to recognize PL’s postulated biopsychosocial health benefits [91], which contribute to its appealing and inclusive nature by emphasizing individual growth throughout the lifespan, regardless of constitution, age, or ability [91]. Consequently, services aligned with PL principles require specific qualifications and skills among practitioners—such as therapists, sports coaches, or PE teachers—to effectively support diverse populations [71]. While there are positions that prioritize performance and quantitative measurement—especially in youth sport and PE contexts—others advocate for a more holistic and harder-to-measure perspective [92]. The broad consensus emerging from these multiple definitions, particularly those propagated by the IPLA and related frameworks, provides a comprehensive and philosophically grounded foundation for advancing PL as a multidimensional and inclusive paradigm that transcends narrow physical competence metrics.

Ultimately, PL is characterized by its holistic nature [40], its flexibility, and its ability to adapt to students and their contexts. It also seeks to foster positive individual experiences for all students, without exclusions, in order to generate a sense of belonging to a group, where each individual is on their own personal journey of PL, and everyone has the capacity to progress according to their own potential and limitations.

### 3.2. Philosophical Foundations of Physical Literacy

**Figure 2:**
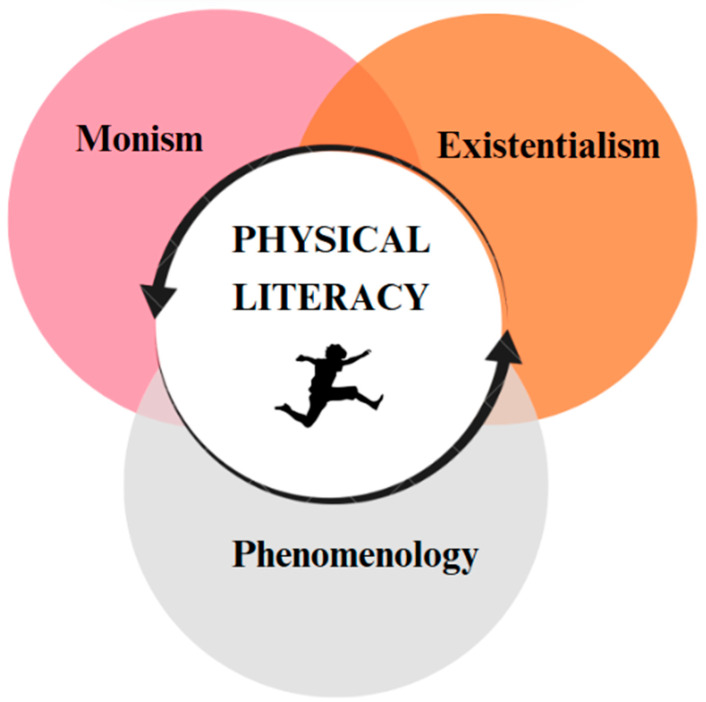
Philosophical foundations of physical literacy.

The concept of PL is grounded in the beliefs of three philosophical currents: monism, existentialism, and phenomenology (Figure 2) [82]. Therefore, it is essential to consider these perspectives when developing authentic teaching that promotes PL. For example, the influence of monism in PL implies that each student should be seen as a whole being, rather than separating mind and body. From the existentialist perspective, this concept is reflected in the need to provide a wide range of situational experiences that allow students to construct their own meaning of learning. Similarly, phenomenology emphasizes that the perception of each situation will be unique to every student, highlighting the importance of addressing individual experiences within the educational process. There are two key factors that support the adoption of PL as a goal in PE: the philosophical foundation of the concept and the elements that make up its definition, which we will address later [39]. Understanding these aspects is essential for designing pedagogical strategies that support the development of meaningful and contextualized PL within the educational setting.

#### 3.2.1. Monism

From the monist perspective, human experience is inseparable from the body and mind, which do not operate as separate entities but as an integrated whole [92]. Individual development occurs through interaction with the context, as this interaction shapes one’s perception and understanding of the world, allowing not only for knowledge of it but also for self-construction within it [82]. Gibbs [93] supports this view by stating that the affective, physical, and cognitive are not separate dimensions, but rather interdependent aspects that form an individual’s identity. Human cognition cannot be understood apart from embodied experience, since knowledge and identity emerge from interaction with the world through the body [93]. Because identity is constructed through embodied experience, teaching and learning processes must acknowledge this interdependence. This means that each individual should be understood in their uniqueness, with a pedagogical approach that respects their holistic development and does not impose a rigid instructional model. Authentic teaching requires empathy and supportive, sensitive feedback, ensuring that students feel valued and motivated throughout their learning process. Therefore, a holistic and monist approach not only enriches the understanding of movement learning but also reinforces the importance of treating each individual with the respect and attention their uniqueness deserves.

#### 3.2.2. Existentialism

From an existentialist perspective, learning is not a passive process but a constant interaction with situations, environments, and other people. Burkitt [94] points out that there is a primordial coexistence between the body and its world, which forms the foundation for the development of awareness and knowledge. The construction of the individual occurs through their interaction with the world, which means that the more diverse and enriching these encounters are, the more fully each person can achieve self-realization. Levins and Lewontin [95] reinforce this idea by affirming that the environment and the organism are mutually related, emphasizing the dynamic and bidirectional relationship between the human being and their context. Similarly, Burkitt [94] emphasizes that the fundamental coexistence between the individual and the world is the basis for the development of consciousness and knowledge. Each learning situation demands an active response from the individual, and it is through this response that opportunities for growth in experience, competence, and appreciation arise. Authentic teaching requires that the educator carefully consider the nature of the situations and the demands to which students must respond. These demands, both in terms of challenge and breadth, should be aligned with the students’ experience and competence in order to promote progressive and meaningful development. Whitehead [82] highlights that this guidance is essential to ensure experiences that match each individual’s potential, allowing them to develop the confidence and physical competence needed to progress on their path toward PL. In this way, learning becomes a process of personal transformation, in which the individual constructs themselves through their ongoing interaction with the world.

#### 3.2.3. Phenomenology

From this perspective, individuals are the result of the cumulative effect of their past experiences, which means that each person perceives, appreciates, and understands a situation or environment through a unique lens. Gallagher [96] explains that this personal perspective is deeply rooted in each individual’s prior experiences, which shape their way of interacting with the world. In the context of authentic teaching, there are three key considerations. First, it is essential to show a sensitive appreciation and genuine respect for each student’s life experiences, recognizing that these shape their learning. Second, the experience of a particular lesson should have a positive impact on the student’s future perceptions, especially in new or challenging contexts within physical competence. Third, since each individual will value the lesson differently, it is crucial to assess its success from the perspective of the learning achieved, rather than through a uniform metric.

Understanding the uniqueness of each student is vital to ensuring that learning experiences promote self-confidence, security, and a positive attitude toward PA. Confidence to engage in new experiences is essential for the progressive development of PL and for fostering the choice of PA as a lifelong commitment. Ultimately, PL involves a set of fundamental skills that enable individuals to navigate their environment with confidence and competence. These fundamental skills encompass:

(a) the development of movement competence, understood as the ability to move with poise, efficiency, and safety across a variety of contexts;
(b) the capacity to overcome physical challenges, demonstrating proficiency and effectiveness in diverse situations; and
(c) the ability to respond appropriately to environmental demands by analyzing and interpreting stimuli in order to make informed decisions [82].

### 3.3. Theoretical Frameworks of Physical Literacy

Our PL model can serve as a key pedagogical framework to promote the holistic development of individuals within the field of PE, addressing not only motor learning but also its affective, social, and cognitive dimensions [82]. In this regard, PL should not be understood solely as a means to improve physical health but as part of an educational model that encourages active participation and the inclusion of all students. To develop this model, three complementary theoretical frameworks have been integrated, allowing for the construction of a solid and coherent proposal. On the one hand, the approach to PL as a determining factor in health promotion and disease prevention has been adopted. This framework views PL as an interconnected set of motor, social, affective, and motivational components essential for an active and healthy life. On the other hand, the theoretical model “PL Praxis” proposed by Gleddie and Morgan [76] has been used as a reference, offering an educational perspective centered on the pedagogical praxis of PL in PE. Additionally, the APLF, recognized as one of the most comprehensive and up-to-date models, has been incorporated to reinforce the pedagogical foundations of our proposal. The combination of these three frameworks supports our model from a multi-faceted perspective: as a pathway to comprehensive health development and as a transformative educational proposal focused on inclusion, sustainability, and meaningful learning.

#### 3.3.1. Conceptual Framework of Physical Literacy as a Determinant of Health

PL is conceived as a key factor in health and disease prevention, mediated through PA and its physiological benefits. Within this framework, PL is understood as an interconnected set of motor, social, affective, and motivational factors [97]. Its cyclical nature highlights the connection between physical competence and emotional and behavioral aspects, promoting participatory and holistic learning [97]. Knowledge is positioned as a result of this cycle of interaction, which in turn can reinforce engagement in PA. This approach emphasizes that motor learning not only enhances physical skills but also strengthens confidence, motivation, and social participation. Furthermore, the convergence of motor, affective, social, and cognitive components is essential in the development of PL. Motor performance alone is not sufficient if it is not linked to positive experiences such as enjoyment and motivation to continue participating in physical and sporting activities [98]. This process is interactive and experienced holistically, without the need for conscious reflection [97]. PA produces physiological adaptations that benefit health, such as increased strength, improved cardiorespiratory fitness, and the release of neurotransmitters, like serotonin [99]. These responses not only improve overall well-being but also reinforce continued participation in PA, completing a cycle of mutual benefits. This model highlights the role of PL as a determinant of PA behavior, distinguishing it from other approaches that consider only motor skills as predictors of PA engagement [100]. In addition, individual factors such as sex, ethnicity, and personality, as well as environmental aspects, like neighborhood and climate, can influence the relationship between PL and PA, as well as the connection between physiological responses and health outcomes [101]. For example, the transition between PL and participation in PA may depend on gender, as certain motor skills are more relevant for girls’ participation than for boys’ [102]. The environment also plays a crucial role: the lack of safe spaces or the presence of socioeconomic barriers can limit participation in PA, reducing its potential benefits [103]. Similarly, physical fitness can moderate the effects of inactivity on health. For instance, good physical fitness may reduce the risk of cardiovascular disease, while low levels of activity can increase that risk [104]. In summary, this model emphasizes the dynamic interaction among PL, PA, and health, underscoring the importance of a holistic and contextualized approach to promoting lifelong well-being.

#### 3.3.2. Conceptual Framework of Physical Literacy in Physical Education

In 2020, Gleddie and Morgan [76], through their theoretical model Physical Literacy Praxis based on the interaction between theory and practice, proposed a PL-centered approach to PE from two main objectives: (1) to equip young people with the tools and knowledge necessary to engage in PA throughout their lives and (2) to ensure that PL is an integral part of the PE curriculum.

Additionally, they address several components: (1) a well-trained educator who is key to establishing a positive learning environment, where meaningful movement experiences drive students’ physical, cognitive, affective, and behavioral development, (2) a form of PL that promotes greater inclusion in PE classes by adapting to each student’s abilities, facilitating their progress, and encouraging a holistic approach that integrates technical, psychological, and social dimensions, (3) a culture that values the principles of PL through movement experiences within learning environments, (4) a quality curriculum that enables pedagogical practices supporting the development and journey of PL, (5) the empowerment of students to become architects of their own learning, developing the knowledge, skills, and attitudes to value and understand movement through meaningful experiences that enhance their holistic development, and (6) four key concepts that interconnect across all components, which are student-centered: motivation and confidence, movement competence, knowledge and understanding, and finally, sustained engagement. In summary, the success of our model will depend on a quality PE program that entails a shift in teaching, adopting a non-linear pedagogy that encourages educators to be intentional in shaping the learning space and in creating an educational climate that values movement as a central experience. When applied within PE curricula, PL will activate a continuous cycle that fosters in students not only motor skills but also the motivation and commitment to maintain a physically active lifestyle throughout their lives.

Therefore, the interrelationship between PE, PA, and PL has become increasingly central in both educational and health-related discourse. Research by Fortnum et al. [105] positions PL as a fundamental determinant of health, mediated by PA and influenced by both individual and contextual factors. Their findings demonstrate that PL is positively associated with key health outcomes, such as aerobic fitness, muscular strength, psychological well-being, and total PA engagement. Similarly, Dlugonski et al. [87] through a systematic review, highlights that PL is not only a predictor of PA participation across the lifespan but also a key outcome of high-quality PE. These results reinforce the role of PE as a unique platform to activate PL, particularly in school-age populations. This is further supported by Houser and Kriellaars [71] who explored how a PL-enriched pedagogy within PE contexts enhances students’ holistic development—cognitively, socially, and affectively—by fostering confidence, motivation, and competence. Their work underscores the potential of quality PE, grounded in PL principles, to generate lifelong engagement in PA by cultivating intrinsic motivation and positive movement experiences. Together, these findings illustrate a dynamic and reciprocal relationship in which PE serves as a structured environment for developing PL, which in turn supports sustained PA participation and contributes to broader health outcomes.

#### 3.3.3. Conceptual Framework Australian Physical Literacy Framework

The APLF, published by Sport Australia in 2016, was developed to address the absence of a unified international understanding of PL and to offer a comprehensive structure for its definition and practical application [53]. This framework emerged following growing concerns about the fragmented conceptualization of PL and the need for a common language among educators, coaches, and policymakers. The APLF proposes a holistic view of PL, defining it as the integration of physical, psychological, social, and cognitive capabilities that enable individuals to move with competence and confidence in a wide variety of physical activities across different environments. It identifies a total of 30 interconnected elements distributed across the four domains, each contributing to the development of a physically literate person. These elements range from physical attributes, such as balance, coordination, and endurance, to psychological aspects, like motivation, self-regulation, and confidence, as well as social skills (e.g., collaboration, ethics) and cognitive functions (e.g., rules, strategy, and decision-making). The APLF also includes a developmental continuum that spans from early childhood to adulthood, allowing for progressive assessment and tailored educational strategies. This broad and inclusive perspective makes the APLF a key reference point for those seeking to embed PL into educational, recreational, or high-performance contexts. Its emphasis on lifelong development, context sensitivity, and the interaction between domains reinforces the notion that PL is not a static goal but an evolving capacity shaped by personal, social, and cultural factors [53].

### 3.4. Principles, Phases, and Elements 

Traditional PE classes have historically prioritized the development of physical fitness, motor skills, and athletic performance [106]. This approach has been focused on activities, such as team sports, fitness tests, and exercises, designed to improve specific physical skills [107]. However, in recent years, the role of PE has evolved, expanding its scope beyond simply engaging in PA. Today, a more comprehensive approach is adopted, one that seeks not only to promote a healthy lifestyle but also to foster individuality, creativity, and the development of communication and cooperation skills. In this context, PE aims primarily to educate physically literate individuals, and teachers play a crucial role in the development and promotion of PL [108]. In this regard, our model of PL will promote the following principles to be developed in PE: (a) promoting holistic learning that develops throughout life, acquired and applied in various movement and PA contexts, (b) reflecting a process of continuous change, integrating physical, psychological, cognitive, and social abilities, (c) leading a healthy and fulfilling life through movement and PA, and (d) achieving the point when an individual is able to integrate their physical, psychological, cognitive, and social abilities to foster movement and PA, promoting health and satisfaction based on their situation and context throughout their life.

This pedagogical model would allow for greater inclusion in PE programs, as well as better retention and progression of students. Additionally, it would represent a shift from a purely technical approach to movement towards a more holistic perspective, integrating psychological and social aspects into the motor experiences of children [109]. The implementation of a pedagogy based on PL within PE classes could be key to activating the cycle of PL for all students. To achieve this, it is necessary to adopt a non-linear pedagogical approach [106], which requires teachers to be deliberate in planning their teaching strategies and in setting up their learning environment. Furthermore, one of the main challenges in pedagogical models applied to PE is the absence of assessment processes that adequately complement learning and encourage student involvement in their own development. Formative assessment, understood as a process that seeks to improve both teaching and learning [110,111], aligns with the principles of PL by providing students with opportunities to reflect on their progress, correct mistakes, and consolidate their knowledge. PE, viewed from this perspective, should not be limited to a series of isolated activities, but rather consider the cumulative impact of all interactions, experiences, and learning throughout the years. The ultimate goal is to leave each student with a unique and positive perspective on PA, fostering an attitude that values continued participation in exercise and movement as an integral part of their life.

In this way, PL is benefited, as constant feedback and self-assessment promote greater awareness among students of their own physical and motor development, which contributes to their motivation and commitment to PA over time [38]. Therefore, integrating these aspects into PE teaching enhances the students’ experience, fostering a deeper understanding of the role that PL plays in their personal development and in their interaction with the world around them [109]. For this reason, the fundamental principles of the PL model for practical implementation are listed below in Table 1 [76,112,113]:

### 3.5. Scientific Publications on Physical Literacy in Physical Education

PL is a fundamental component of PE, as it fosters the competence, confidence, and motivation required for lifelong engagement in PA. Numerous studies have evaluated the impact of programs designed to enhance PL among students. For instance, the “Run, Jump, Throw” (RJT) program, implemented with 111 students and compared to 76 peers who received regular PE instruction, led to significant improvements in motor competence after eight weeks. These improvements were assessed using the PLAYfun tool (*p* < 0.01 for time; *p* < 0.05 for group interaction) [114]. Similarly, Kriellaars et al. [115] incorporated circus arts into PE classes for fourth- and fifth-grade students, resulting in notable gains in PL, thereby positioning circus arts as an effective pedagogical tool. Mandigo et al. [116] evaluated an eight-week extracurricular program based on the Teaching Games for Understanding (TGfU) model with 22 primary school children. The results showed significant improvements (*p* < 0.004) in balance, stability, cardiovascular endurance, and participation in various environments, although no significant changes were observed in kicking skills or social interaction. In another study, Farias et al. [117] investigated the long-term effects of a year-long Sports Education program among secondary school students. Three years post-intervention, 24 participants reported positive developments in their PL, including increased empathy, resilience, and a commitment to equitable participation in both PE and sports contexts. Invernizzi et al. [118] assessed a pedagogical approach combining multiple teaching styles with active reflection among 121 primary school students. After 12 weeks, the intervention group demonstrated significant improvements in physical fitness, motor competence, enjoyment, and PA levels, alongside increased engagement and satisfaction with PE classes. Lastly, Stoddart et al. [119] implemented a Physical Literacy in Physical Education (PLitPE) program with 131 primary students. The intervention yielded significant gains in motor competence (M = 49.4 vs. M = 40.0; *p* < 0.001) and movement vocabulary (M = 10.3 vs. M = 7.5; *p* < 0.001), while also reducing gender disparities. However, no significant differences were found in PA participation levels. These findings underscore the value of high-quality, well-structured PE programs in fostering PL among children and adolescents. Improved PL not only enhances motor competence but also contributes to broader health and well-being outcomes, encouraging sustained participation in physical activity throughout life. Consequently, integrating effective pedagogical approaches into school PE is essential for promoting healthy and active lifestyles from an early age.

## 4. Discussion

PL is grounded in a clear and close relationship between learning, teaching, subject matter, and context [18], as well as in understanding the rationale and purpose of a model, as established by various authors [113]. Findings from the narrative review highlight the origins and historical evolution of the PL concept, emphasizing its relevance to human embodiment since ancient times. In this context, the philosophical foundations supporting the PL model are rooted primarily in traditions, such as monism, existentialism, and phenomenology, which have contributed to the development of contemporary conceptual frameworks linking PL with both education and health, especially highlighting its connection with PA and well-being. Furthermore, several key pedagogical elements have been identified as essential for fostering the development of PL in PE classes. These include: qualified teachers, empowerment, connection to the place, self-regulation, society and culture, collaboration, fun, creativity, consistent engagement, session models, inclusion, time perspective, and evaluation. In addition, various PL-based interventions have been shown to effectively enhance the development of PL in school settings. Based on these findings, we propose an emerging model of PL for PE that integrates the following key aspects: (a) determining appropriate methodologies for the effective development of the model; (b) positioning PL as an inclusive right for all students; and (c) promoting its development from early childhood.

### 4.1. Physical Literacy: An Emerging Pedagogical Model

**Figure 3:**
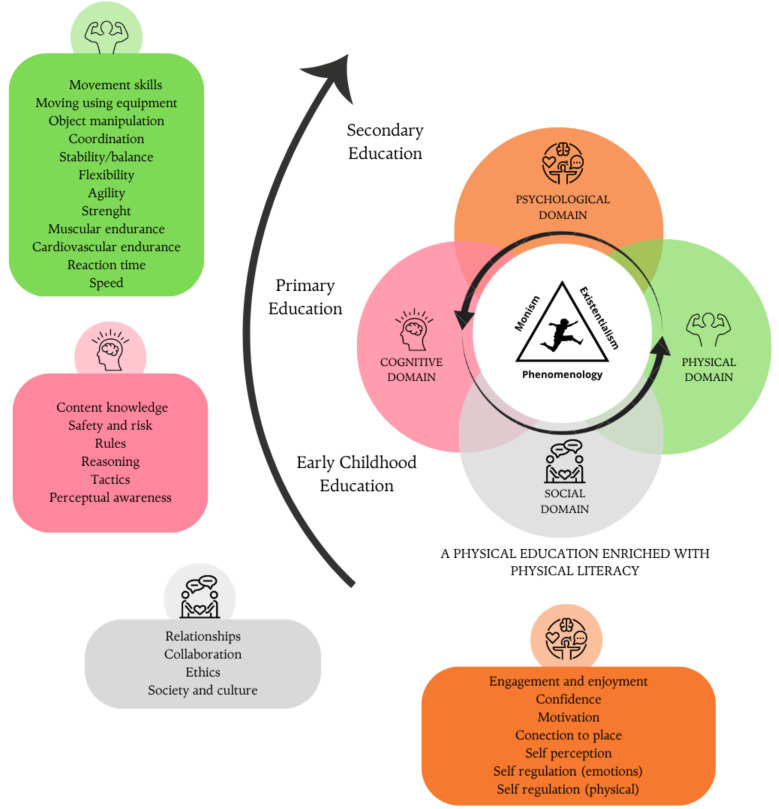
The development of our emerging model of physical literacy.

In Figure 3, the development of our emerging model of PL is presented, based on the results found, which highlight the philosophical roots, key pedagogical elements, and effective strategies necessary to promote PL in PE. In this model, the student occupies a central role as the architect of their own learning, developing knowledge, skills, and attitudes that allow them to value and apply the principles of PL. The teacher, in turn, acts as a guide in this process. To achieve this, it is essential to emphasize the interrelationship between the mind, body, and environment in the development of the individual, with the goal of reaching a philosophical understanding of PL. From the perspective of monism, the mind and body are interdependent entities, integrated into a unified and conscious self [107]. According to this view, the experience of the world is not fragmented, but rather a continuous process in which the individual is shaped through their interaction with the environment. The greater this interaction, the greater the personal development, as it enriches the perception and understanding of the world. This principle is closely related to existentialism, which posits that the formation of the self depends on the constant relationship with the external world [82]. Since the environment is dynamic and changes independently of the individual’s presence, each experience is unique and contributes to the construction of identity and personal development. On the other hand, phenomenology explains that the perception of the world is influenced by past experiences. Our current understanding is the result of the accumulation of past experiences, shaping a unique perspective of reality [107].

In this framework, and to complete a Quality Physical Education (QPE), our PE sessions must explicitly connect with the four interrelated domains of PL: physical, psychological, cognitive, and social. Within these domains, 30 specific elements are identified that can help structure a class session, as outlined in the APLF, which articulates 30 interconnected elements across multiple domains and is currently considered one of the most comprehensive models available [113]. For example, the physical domain includes movement skills, the psychological domain encompasses motivation, the cognitive domain integrates knowledge of rules, and the social domain emphasizes collaboration. Each of these elements is essential for the formation of physically literate individuals, providing a comprehensive view of motor, emotional, cognitive, and social development in the context of PA.

#### 4.1.1. Physical Domain

Gross motor competence and PA have a reciprocal relationship, where children with greater competence in movement skills tend to participate more in physical activities, reinforcing their motor development, while those with less competence tend to engage less, perpetuating a cycle of inactivity [120]. Studies have shown that higher levels of PL are associated with greater adherence to PA guidelines and improvements in cardiorespiratory health [121]. Furthermore, the perception of one’s motor competence influences motivation and commitment to PA. Children who feel skilled in movement show greater willingness and enjoyment in physical practice, while negative perceptions can demotivate them [122]. Beni et al. [123] highlight that motor competence not only facilitates participation but also influences the understanding of one’s performance and the response from the social environment, emphasizing the importance of a positive learning environment. From a pedagogical perspective, students arrive at PE with prior skills that influence their learning. The implementation of structured practices, such as gymnastics activities and strength training, promotes postural stability, locomotion, and coordination [124]. Additionally, innovative strategies based on play and performing arts have promoted a more holistic development, moving away from the traditional performance-based approach and moving towards an intrinsic appreciation of movement [82]. For example, using the slackline, where students must maintain dynamic balance on a tensioned rope, which requires postural control, core activation, and intermuscular coordination. Another option would be to incorporate adapted parkour movements, such as precision jumps, climbing, or agile movements, which develop power, agility, and control of the body in space. Or a circuit of stations focused on functional exercises, like push-ups, squats, or lunges, performed in high-intensity sequences that challenge muscular strength, endurance, and joint stability.

#### 4.1.2. Cognitive Domain

PL not only involves motor development but also a strong cognitive component that allows individuals to understand and apply movement throughout life [125]. Processes, such as understanding, communication, application, analysis, and strategic thinking, are essential for decision-making in various physical activities, from games to dance and outdoor sports. This emphasizes the importance of a movement vocabulary aligned with other academic disciplines, reinforcing the cognitive aspect of PE. Declarative and procedural knowledge is key to motor performance and in pedagogical models, such as Teaching Games for Understanding, where strategy and decision-making improve participation and physical fitness [126]. According to Hattie [127], metacognition allows students to self-regulate their learning, fostering their autonomy and commitment to an active lifestyle. Teachers should use strategies that integrate cognitive learning with PL, such as sports education and decision-making-based models [128]. An example is the “movement on wheels,” where students choose their mode of transportation (scooter, bicycle, rollerblades) in a structured environment. Another example is bouldering, which prioritizes problem-solving over achieving specific levels, allowing each student to face challenges adapted to their ability. These strategies reinforce creativity, autonomy, and commitment to PA both inside and outside the school environment.

#### 4.1.3. Psychological Domain

The psychological domain of PL highlights the influence of emotions and attitudes on personal development, where motivation is key during childhood [53]. Strategies, such as cooperative learning or the use of other pedagogical models related to PL, foster connections between students and strengthen their confidence and perceived competence [129]. Furthermore, allowing them to track their own progress and offering opportunities for additional practice creates a motivating environment tailored to each skill level [130]. Setting progressive challenges helps transform initial emotions, such as anxiety, into achievement experiences, promoting resilience, and valuing effort [130]. One example of this is the design of challenge circuits in teams, where students overcome progressively difficult stations through cooperation. This approach strengthens intrinsic motivation, encourages persistence in the face of challenges, and promotes learning based on mutual support. Likewise, authentic relationships between teachers and students are essential for students to perceive themselves as active agents in their learning [129]. Progressing in their skills generates enjoyment and strengthens their positive bond with PA, increasing the likelihood of maintaining an active lifestyle over time [53]. For example, a class can be organized with a cooperative team circuit, where each station increases in difficulty. Teams move forward only when all members complete the task, promoting empathy, mutual support, and resilience. Another option is a body dramatization activity, where students express emotions through movement, strengthening emotional intelligence and connection to PA. Cooperative learning with assigned roles (such as motivator or assistant) can also be used, giving each student an active role that builds a sense of belonging and confidence.

#### 4.1.4. Social Domain

The social aspect in PE plays a key role in building relationships and promoting participatory learning, with collaboration being highlighted as an essential element [53]. Qualitative evidence indicates that teamwork and positive interactions within the classroom facilitate this learning, as they allow students to exchange ideas and reflect on their learning process [131]. Moreover, belonging to a social circle and interacting with others have been shown to be determining factors in promoting movement behaviors [132]. From a PL approach, enriched pedagogical practices can foster positive movement experiences in PL, preventing the accumulation of negative experiences. These practices include designing activities with challenge levels adapted to all abilities, developing genuine connections with the environment and others, fostering teamwork and empathy, and promoting creative movement [132]. Additionally, teachers should emphasize strategies focused on developing physical, social, and psychological competencies, such as peer teaching (cooperative learning) and mitigating the fear of failure by intentionally building students’ confidence. For example, the co-operative strategy “pairs check and perform” can be used, where one student performs a movement (like push-ups) while the other observes, gives feedback, and encourages. Then, they switch roles, promoting empathy and peer learning. A collective score system can also be used, where all students contribute to a shared goal (such as 200 squats), encouraging cooperation and reducing individual pressure.

In short, the proposed PL model seeks a holistic contribution to each PE session, ensuring the comprehensive development of students across the physical, cognitive, psychological, and social domains, with each domain consisting of a series of elements that can be applied in the class based on the Australian Framework. Furthermore, it is grounded in a series of key principles, such as the presence of a qualified teacher capable of guiding the process with effective pedagogical strategies, empowering students to foster their autonomy and confidence, connecting with the place by integrating learning with the environment, as well as self-regulation, understanding society and culture, and the importance of collaboration, all of which reinforce active participation by students in their learning process. Elements, such as fun, creativity, and constant engagement, are essential for maintaining motivation and interest in PA, while a structured session model, the inclusion of all students, an appropriate time perspective, and rigorous evaluation ensure that learning is meaningful and progressive, based on triadic evaluation processes: self-assessment, peer assessment, and teacher assessment, allowing for consensus in the final grading through a shared evaluation process and dialogued grading [133]. Finally, the general arrow in the center of the model emphasizes the dynamic and evolving nature of PL, indicating that its development in the early stages of life influences future participation in PA and the associated benefits throughout life. Thus, it reinforces the idea that PL is not a destination but rather a continuous and lifelong journey.

### 4.2. Methodology for Physical Literacy

The analysis of 21 research articles allows us to conclude that teacher-centered pedagogy, also known as linear pedagogy, has a limited impact on the improvement and promotion of PL [134]. Studies indicate that students exposed to this approach are less likely to develop awareness and understanding of the importance of health knowledge and lack reflective thinking skills [135].

On the other hand, non-linear pedagogy has shown significant influences on children’s PL, favoring content knowledge, self-determination, self-regulation, perceived motor competence, self-efficacy, and physical engagement [136]. Some studies also suggest that the combination of linear and non-linear pedagogy in PE generates a broader positive impact on PL, strengthening content knowledge, functional motor skills, and motivation for an active lifestyle [137]. Furthermore, the comparison between performance-based and competence-based pedagogies reveals key differences. Performance-based PE promotes enjoyment of movement, self-directed learning, and problem-solving skills [138]. In contrast, competence-based pedagogy strengthens children’s confidence and competence in PA, facilitating their interpersonal relationships and promoting self-awareness in health and well-being beyond sportsmanship and achievement [139].

Among these pedagogies, Teaching Games for Understanding (TGfU) is one of the most widely adopted strategies. Studies have demonstrated its positive impact on motor skills, technical and tactical knowledge, physical performance, and personal development, including collaboration, decision-making, and reflective thinking [140]. This approach, developed by Almond along with Bunker and Thorpe [141], has been recognized as an effective model for promoting PL [72]. Its success is due to its alignment with the fundamental principles of PL. Mandigo and Corlett [142] highlighted its impact on knowledge acquisition, technical and tactical skills development, and generating positive motivation. Later research supports its effectiveness in skill execution, tactical transfer, motivation, and decision-making in games [143]. TGfU is based on four pedagogical principles aligned with PL: sampling, which allows for the transfer of skills and tactics between similar games; representation, which reflects the actual use of skills, rules, and tactics; exaggeration, which highlights specific aspects of the game; and tactical complexity, which gradually increases the difficulty of the game through strategic questions [144]. These principles are interconnected with the physical, cognitive, psychological, and social domains of PL, consolidating TGfU as a key tool for fostering active participation, autonomous decision-making, and intrinsic motivation in students. The same applies to the sports education model, which aims to develop students’ competence in forms of play, establish literacy through knowledge of rules and practice, and enhance motivation toward PA and sports [145], thus facilitating all domains of PL, including motor, cognitive, social, personal, and emotional development, models that can be hybridized with the PL model to achieve the holistic development of students.

Other pedagogical strategies, such as daily seminars and paper-based information distribution, have shown limited impact, focused on improving PL from knowledge. These strategies help children understand the importance of PA, a healthy lifestyle, and proper nutrition [146,147]. However, the integration of health education into regular classes has shown deeper influences on PL, facilitating the articulation of health knowledge, a higher level of participation in PA, and better application of this knowledge in daily life [148]. Finally, among the countries adopting more systematic and evidence-based pedagogies are Italy, England, the United States, Australia, Ireland, Sweden, and Spain. These countries implement methodologies, such as TGfU, inquiry-based learning, the Health Belief Model, non-linear pedagogy, and Invisible Pedagogies, consolidating a more structured approach to PL, which could be used to foster or develop the PL model [144].

### 4.3. Inclusion: Physical Literacy for All

In today’s society, the media and press tend to focus on high-performance sports, international competition, and the impressive physical feats of sports stars [107]. Additionally, they promote the development of extreme sports, reinforcing the idea that only these exceptional physical abilities deserve recognition. As a result, many people come to perceive themselves as physically incompetent when compared to these unattainable standards [107]. Phrases like “I’m not good at sports” become common, leading to a lack of motivation to participate in physical activities. This misconception can lead to a loss of confidence in one’s abilities and abandonment of movement as a source of satisfaction and well-being [107]. However, every person possesses the fundamental elements of PL. This ability is not limited to high performance but is a potential present in every individual, ready to be developed through meaningful experiences in appropriate contexts [82].

Each person embarks on a unique path in their PL journey, and progress should not be measured against universal standards but rather in relation to one’s own personal development. PL is a right for all, regardless of their abilities or the cultural environment in which they live. It is not about reaching an unattainable ideal but about exploring and strengthening the relationship with movement in an autonomous, enjoyable, and inclusive manner [107]. The physical dimension is an essential aspect of human existence. We live and understand the world through the body and its interaction with the environment. In this sense, PL is not just a matter of motor competence but also of confidence, self-awareness, and overall well-being [107]. Developing this ability allows individuals to enjoy multiple benefits, both physical and emotional, as well as social. Nevertheless, debates persist over whether PL is truly achievable for everyone or if it is reserved for those with greater physical abilities [82]. This text argues that everyone can develop it, although its expression may vary according to each person’s abilities and contexts [107]. The key is to promote accessible and inclusive opportunities, avoiding that the success model imposed by the media demotivates or excludes those who do not identify with it.

In Western culture, there has been a built-in attitude of rejection toward physical ability, leading many individuals to avoid PA due to fear of failure or embarrassment. The media reinforce this mindset by establishing unattainable performance models [107]. Therefore, it is urgent to redefine PL as a universal and accessible right for everyone, promoting an inclusive and encouraging vision where each person can discover and develop their physical potential. PL encompasses the skills, attitudes, and habits necessary to actively participate in a culture of movement throughout life [82]. From a holistic and inclusive approach, it promotes sustained participation in physical activities and encourages healthy lifestyles for the entire population, including people with disabilities. However, despite the efforts of various initiatives and organizations, barriers persist that perpetuate an ableist view of movement, limiting real inclusion. To move towards true equity in PL, it is crucial to adopt a sociocritical perspective that values bodily diversity and recognizes the right of all individuals to participate in PA without being judged or marginalized. Only through an inclusive approach, which appreciates individual differences and adapts movement opportunities to each person, will we transform the dominant narrative and build a society in which PL is truly for all.

### 4.4. Development from Early Ages and Its Evolution

Each of the fundamental elements of PL, such as motivation, confidence, physical competence, knowledge, and understanding, can be cultivated throughout life. During childhood, these key elements establish an essential foundation for the development of PL, as growth during this stage involves not only changes in the body (e.g., in the skeletal, muscular, and nervous systems) but also the strengthening of fundamental aspects of movement. In this context, PL is structured around three essential components: the movement vocabulary, which encompasses a variety of motor skills, such as balance and locomotion (crawling, climbing, jumping, running, among others), movement memory, which refers to the ability to internalize movement experiences, and the quality of movement, which involves performing movements with precision, coordination, and control [148]. Play, as an activity inherent to child development, plays a key role in enhancing these skills, stimulating not only physical development but also cognitive, social, creative, and imaginative development. It has been shown that a combination of free play—where children explore and experience movements spontaneously—and guided play—facilitated by adults with knowledge to promote challenges and structured learning—fosters the enrichment of PL. These games can take place in various environments and with different resources, both natural and artificial, in order to maximize the development of these abilities [148].

Furthermore, the influence of significant people in children’s lives plays a determining role in the development and maintenance of PL. During childhood, parents, caregivers, and teachers have a crucial impact, as their constant interaction with children allows them to shape positive attitudes toward PA [107]. Encouraging enjoyment in the context of PA is essential for strengthening PL. To achieve this, it is important for these adults to not only motivate children to participate but also actively involve them in the planning of activities and provide guidance in a supportive environment. It is key that movement skills are taught in meaningful contexts and not in isolation, thus minimizing any feelings of incompetence in children. Although opportunities to participate in PL vary throughout the life cycle, empathetic encouragement and motivation remain essential at all stages of development [107]. Within this framework, teachers and coaches have a key responsibility in the development of children’ PL. In the school context, teachers can provide structured instruction and constructive feedback that favors safe and effective motor development. In particular, PE teachers possess the specialized knowledge needed to analyze and break down complex movements, which helps children understand and refine their physical skills. They also have the opportunity to promote inclusion, ensuring that all students actively participate, and to serve as role models who inspire and motivate children to stay active. Through intentional teaching adapted to the needs of each student, teachers can strengthen the development of PL from childhood, thus laying the foundation for an active and healthy life.

## 5. Conclusions

The importance of PL has been highlighted as an integral process that encompasses the development of motor, psychological, cognitive, and social skills necessary to actively participate in movement and PA throughout life. Its application in different contexts, adapted to the students, linked to any content, and the learning that is intended to be acquired, allows it to be affirmed as an emerging pedagogical model due to its usefulness in helping teachers improve their educational practice with the goal of achieving quality PE. This can be achieved by using different elements drawn from the four domains that make up PL, as an interrelation between the components of these domains in a PE session will contribute to the development of PL, and in turn, to the development of quality PE. Furthermore, our PL model will promote the development of holistic learning that will evolve throughout life, being acquired and applied in various contexts of movement and PA. This holistic process requires the interrelation of physical, psychological, cognitive, and social capacities for the individual to achieve a healthy and fulfilling life through movement and PA.

The examples presented reflect how PE can be structured to foster this literacy, ensuring that students develop their abilities in different domains and are capable of transferring those learnings to their daily lives. Moreover, the need to continue exploring teaching methodologies that promote active participation and meaningful learning is evident, ensuring that individuals acquire and maintain movement habits that allow them to enjoy an active and healthy life. Therefore, PL should be understood as an emerging pedagogical model that allows for the integral structuring of PL teaching. Its application in various educational contexts, along with appropriate formative assessment, can enhance teaching practices and ensure that students develop the necessary skills to stay active throughout their lives. In this regard, future research should focus on consolidating pedagogical strategies that strengthen PL in various environments, ensuring its positive impact on the holistic development of individuals and their long-term quality of life.

## 6. Limitations

It is important to acknowledge the inherent limitations of this study. Although a structured literature search was conducted, this review is narrative in nature, which may limit objectivity and reproducibility compared to systematic reviews. No formal assessment of quality or risk of bias was carried out, as this is not customary in narrative reviews. Additionally, the selection and interpretation of studies may have been influenced by the authors’ perspectives, and some relevant works may not have been identified due to the inherent limitations of the search strategies employed.

## Figures and Tables

**Table 1:** The fundamental principles of physical literacy.

Principle	Description
Qualified teacher	The importance of having the PE program taught by an educator who possesses the knowledge, understanding, and skills necessary to deliver a well-planned and purposeful program
Empowerment	Students should be the architects of their own learning, developing the knowledge, skills, and attitudes to value and embody the principles of PL, including persistence, independence, initiative, and the motivation to continue their journey.
Connection to the place	Appreciation and connection with the environment, both built and natural, in relation to movement and PA.
Self-regulation	The ability to manage emotions and behaviors related to movement and PA.
Society and culture	Appreciation of the cultural values present in groups, organizations, and communities, such as organizing group activities, teaching cooperative play, promoting friendship, and accepting diversity.
Collaboration	Social skills for successful interaction with others, including communication, cooperation, leadership, and conflict resolution.
Fun	Tasks adapted to the students’ skill level that foster intrinsic motivation, high participation, and skill development.
Creativity	Inspiring innovation, fostering problem-solving, accepting mistakes, and allowing young people to create new games.
Consistent engagement	Development of regular practice, emphasizing the importance of an active lifestyle throughout life.
Session model	Achieving the comprehensive development of students in a single session through the physical, cognitive, psychological, and social domains. The elements of the model (Figure 2) will be taken into account for this.
Inclusion	The individual experience of the student should be the starting point for promoting PL, where each student is unique, with specific skills, preferences, and experiences that distinguish them. Each individual is on their own personal journey of PL, and all have the potential to progress according to their own abilities and limitations.
Time perspective	PL is understood as a lifelong journey.
Evaluation	Of a formative and shared nature.

## Data Availability

Not applicable.

## Abbreviations

The following abbreviations are used in this manuscript:

PL	Physical Literacy
PA	Physical Activity
PE	Physical Education
